# VRK1 functional insufficiency due to alterations in protein stability or kinase activity of human *VRK1* pathogenic variants implicated in neuromotor syndromes

**DOI:** 10.1038/s41598-019-49821-7

**Published:** 2019-09-16

**Authors:** Elena Martín-Doncel, Ana M. Rojas, Lara Cantarero, Pedro A. Lazo

**Affiliations:** 1 Molecular Mechanisms of Cancer Program, Instituto de Biología Molecular y Celular del Cáncer, Consejo Superior de Investigaciones Científicas (CSIC) - Universidad de Salamanca, Salamanca, Spain; 2 Instituto de Investigación Biomédica de Salamanca (IBSAL), Hospital Universitario de Salamanca, Salamanca, Spain; 3 Centro Andaluz de Biología del Desarrollo (CABD), CSIC-Universidad Pablo de Olavide, Sevilla, Spain; 4 Instituto de Biomedicina de Sevilla (IBIS), CSIC-Universidad de Sevilla, Hospital Universitario Virgen del Rocío, Sevilla, Spain; 5 Laboratorio de Neurogenética y Medicina Molecular, Institut de Recerca Sant Joan de Déu, Esplugues de Llobregat, Barcelona, Spain

**Keywords:** Kinases, Nucleoproteins, Mechanisms of disease, Neurodevelopmental disorders

## Abstract

Very rare polymorphisms in the human *VRK1* (vaccinia-related kinase 1) gene have been identified in complex neuromotor phenotypes associated to spinal muscular atrophy (SMA), pontocerebellar hypoplasia (PCH), microcephaly, amyotrophic lateral sclerosis (ALS) and distal motor neuron dysfunctions. The mechanisms by which these VRK1 variant proteins contribute to the pathogenesis of these neurological syndromes are unknown. The syndromes are manifested when both of these rare *VRK1* polymorphic alleles are implicated, either in homozygosis or compound heterozygosis. In this report, to identify the common underlying pathogenic mechanism of *VRK1* polymorphisms, we have studied all human VRK1 variants identified in these neurological phenotypes from a biochemical point of view by molecular modeling, protein stability and kinase activity assays. Molecular modelling predicted that VRK1 variant proteins are either unstable or have an altered kinase activity. The stability and kinase activity of VRK1 pathogenic variants detected two groups. One composed by variants with a reduced protein stability: R133C, R358X, L195V, G135R and R321C. The other group includes VRK1variants with a reduced kinase activity tested on several substrates: histones H3 and H2AX, p53, c-Jun, coilin and 53BP1, a DNA repair protein. VRK1 variants with reduced kinase activity are H119R, R133C, G135R, V236M, R321C and R358X. The common underlying effect of VRK1 pathogenic variants with reduced protein stability or kinase activity is a functional insufficiency of VRK1 in patients with neuromotor developmental syndromes. The G135 variant cause a defective formation of 53BP1 foci in response to DNA damage, and loss Cajal bodies assembled on coilin.

## Introduction

Understanding the pathogenesis of neurodegenerative diseases is a major medical challenge, which is needed to improve their early diagnosis or development of protocols to control or better the life conditions of affected individuals. Recently, several complex neurodevelopmental, and neuromotor syndromes, have been associated to rare alleles of the human *VRK1* gene. VRK1 is a chromatin serine-threonine kinase that regulates different nuclear proteins. VRK1 directly regulates chromatin remodeling by phosphorylation of histones H3^1–3^ and H2AX^4^, and indirectly by the acetylation of histones^2^. It also phosphorylates several transcription factors such as p53^5,6^, c-Jun^7^, ATF2^8^, CREB^9^, Sox2^3^ and the farnesoid X nuclear receptor HR1H4 in its DNA binding domain^10^. VRK1 also regulates proteins implicated in different steps of DNA-damage responses (DDR) such as H2AX^2^, NBS1^11^ or 53BP1^12^. These phosphorylation targets implicate VRK1 in several cellular functions such as the regulation of cell cycle progression and cell division^13,14^, mitosis^1,3^, regulation of transcription and of DNA damage responses^2,11,12,15^ and DNA repair^16^. Other processes that are regulated by VRK1 are telomere lengthening^17^, Golgi fragmentation^18^, Cajal bodies (CB)^19^ and nuclear envelope dynamics^20^ in mitosis. Therefore, all these functions are likely to be defective in patients with *VRK1* pathogenic variants. Mutations in genes associated to DNA repair processes are frequently manifested as neurodevelopmental syndromes^21^.

The homozygous R358X pathogenic variant was associated to a spinal muscular atrophy (SMA) and pontocerebellar hypoplasia type 1 (PCH1)^22^. This *VRK1-*R358X variant codes for a truncated protein that is unstable and mislocalizes to the cytosol^23^. Another patient also with homozygous R358X variant^24^, both parents are carriers, presented a related phenotype with microcephaly and peripheral neuropathy with secondary muscular atrophy, but their presentation is quite distinct owing to the lack of PCH on MRI, lack of central nervous system neurological symptoms (ataxia, hypertonia), and normal cognitive function^24^. Other VRK1 variants have been associated to complex motor and sensory axonal neuropathies. Two sisters having compound heterozygous variants V236M and R89Q, each parent carries one of the variants, presented a delayed neuromotor development and microcephaly with normal cognition^24^. Other mutations are associated with early onset amyotrophic lateral sclerosis^25^. Compound heterozygous missense mutations H119R and R321C were detected in a case with adult-onset, distal lower extremity predominant, progressive weakness with upper and lower motor neuron signs, probable ALS^25^. The pathogenic variant R133C was detected in a case of cognitive disorder^26^. Additional cases with a deletion proximal to the *VRK1* gene have been reported in several members of a family, but it is not known whether the *VRK1* gene expression was altered^27^. The heterogeneity of these pathogenic variants and the neurodevelopmental defects, although all are distal neuromotor problems, suggested that VRK1 is underlying some basic functions in the affected neurons by unidentified mechanisms.

In this work, we have biochemically characterized the human VRK1 pathogenic variants associated to neuromotor and neurodevelopmental phenotypes in order to detect their mechanistic contribution to the pathogenesis of these syndromes.

## Results

### Phenotype and frequency of human *VRK1* mutations associated to neurological syndromes

The pathogenic heterogeneity of human *VRK*1 variants is shown in Table 1. In order to manifest the clinical phenotype, both two alleles need to be variants. The neurological phenotypes were detected in patients having either homozygous or compound heterozygous variants. The parents are carriers of a heterozygous variant recessive allele. The frequency of the known human *VRK1* pathogenic variants was searched in the Genome Aggregation Database^28^ (Table 2). All human VRK1 pathogenic variants are very rare alleles in the general population, and some of them are often detected in the Ashkenazi population.

**Table 1:** Heterogeneity of neurological phenotypes associated to human *VRK1* pathogenic variants.

Patient information	VRK1 pathogenic variants							
	R358X R358X	R358X R358X	R358X R358X	R358X H119R	H119R R321C	R133C R133C	R89Q V236M	G135R L195V
SMA	Distal	—		Distal	Distal	No	Distal	Distal
Clinic/MRI	SMA-PCH1	cortical dysplasia	simplified gyral pattern, and vermian hypoplasia	Sensory-motor axonal neuropathy	ALS	cognitive disorder	Sensory-motor axonal neuropathy	Juvenile ALS?/Sensory-motor axonal neuropathy
PCH	Yes	No	No	No	No	No	No	No
Microcephaly	Yes	Yes	Yes	No	No	No	Yes	Yes
ataxia	Yes	Yes	No	No	No	No	No	No
Mental deficiency	Yes	Yes	No	No	No	Yes	No	No
origin	Ashkenazi	Ashkenazi	Ashkenazi	Ashkenazi	Hispanic	Iranian	European	Unknown (USA)
Age	Childhood	Prenatal	Prenatal	Adult	Adult	Childhood	Childhood	Childhood-adult
No. cases	2	1	1	2	1	1	2	1
Reference	^22^	^88^	^24^	^46^	^25^	^26^	^24^	^46^

**Table 2:** Frequency of *VRK1* (OMIN 602168) pathogenic variants in the Genome Aggregation Database (genomAD)^28^.

VRK1 pathogenic variant	Allele Frequency^28^	ClinVar (allele ID)	Presentation of recessive variants
**R89Q** (rs773138218)	3.19e-5	197213	Compound heterozygous
**H119R** (rs371295780)	2.83e-5	209204	Compound heterozygous
**R133C** (rs387906830)	1.2e-5	30243	Homozygous
**G135R**	*de novo* (?)	—	Compound heterozygous
**L195V** (rs748878251)	7.96e-6	533534	Compound heterozygous
**V236M** (rs771364038)	2.39e-5	218924	Compound heterozygous
**R321C** (rs772731615)	1.95e-4	209205	Compound heterozygous
**R358X** (rs137853063)	6.39e-5	7497	Homozygous & Compound heterozygous

### Structural alterations and modelling of the human VRK1 pathogenic variants in neuromotor syndromes

**Figure 1:**
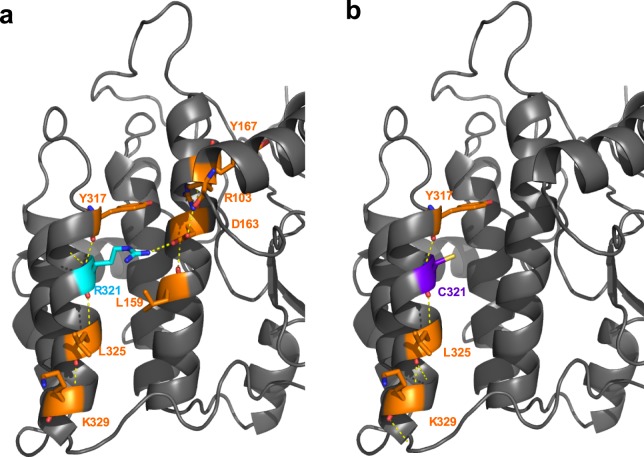
Structure of VRK1 destabilizing R321C mutation and the interaction network of residues via Hydrogen bonds. (**a**) Wild type interaction network is R321, D163, Y317, L325, K329, Y167, R103, and L159. (**b**) The VRK1 variant C321 is destabilizing and loses a significant part of this interaction network, C321, Y317, L325, K329. When polar interactions are present within the interacting residues, they are indicated by yellow lines. In the graphic, the protein is represented as a gray cartoon. The WT residue is cyan, the mutated residue is purple. When polar interactions are present within the interacting residues, yellow lines indicate them. The interacting residues are orange.

To detect the possible structural effect on the stability of the VRK1 human pathogenic variants, these variants were modelled using the known three-dimensional structures of VRK1 in the Protein Data Bank using the X-crystal (2RSV)^29^ and nmr (2LAV)^30^. These structures lack the C-terminal low complexity region that has alternative foldings^30^. The location of the different pathogenic variant aminoacids on the structure indicate that topologically they are in very different regions of the protein (Supplementary Fig. S1). To check whether the identified pathogenic variants of VRK1 have an effect on the structural stability of the kinase, we analyzed them using the empirical FoldX forcefield method^31^. In this method, the stability of a protein is defined by the changes in free energy (in Kcal/mol), the lower the value, the more stable is the protein. In general, a variant that brings free energy changes (ΔΔG > 0 kcal/mol) will destabilize the structure (Supplementary Table 2). The differences in free energy caused by each pathogenic variant were similar in all the structures available (Table 3). The analysis of structural changes predicts that pathogenic variants G135R, R321C and L195V have a destabilizing effect (Table 3). These variant aminoacids alter the interaction network of these residues. In the case of the R321C variant, this R321 interacts with a neighboring helix through D163 and its folding is disrupted by its change to cysteine (Fig. 1). There are also disruptions with the G135R variant (Supplementary Fig. S2), the variant showing a higher energy difference, and that has novel interactions with N186, Y187 and L185. Residues R133 and G135 are located in the interaction region with the adenine moiety of ATP^30^. The R89Q (Supplementary Fig. S3), H119R (Supplementary Fig. S4), and R133C (Supplementary Fig. S5) variants have no apparent effect on the stability of the kinase domain, but have a flexible organization with alternatives rotamers, which may condition their interaction or accessibility to regulatory proteins. The V236M variant does not show any detectable change in interactions within the protein, but may alter its interactions with other proteins or modulators (Supplementary Fig. S6). The R358X variant, is a truncated protein that lacks its C-terminal region and the nuclear localization signal, mislocalizing to the cytosol^23^. However, the folding of the C-terminal of VRK1 regulates the kinase activity^30^ and thus this R358X variant has only a residual kinase activity^23^, which was confirmed in autophosphorylation and transphosphorylation assays of several substrates. The flexible VRK1 C-terminal region, which was not included in the X-ray structure, regulates the kinase activity^30^. Therefore, effects of some variant proteins may be the consequence of interactions with this low-complexity and flexible regulatory region.

**Table 3:** Prediction of VRK1 pathogenic variant protein stability.

Pathogenic variant	Structure used and difference in total energy from wild type and VRK1 pathogenic variant proteins			Stability prediction
	2RSV_diff (2018)	diff_2RSV	diff_2LAV	
G135R	2,4208	2,9714	0,6761	destabilizing
H119R	−0,8401	−0,7466	−1,5024	stabilizing
L195V	0,1785	0,0108	1,5525	destabilizing
R133C	−0,7308	−0,783	−0,6514	Light stabilizing
R321C	1,7191	1,7558	0,725	Light destabilizing
R89Q	−0,1242	−0,065	−0,72	stabilizing
V236M	0,3835	0,3926	−0,3905	neutral
R358X	Not applicable: truncated protein			

Differences in total energy between wild type and each VRK1 pathogenic variant proteins, and prediction of the effect on their stability. The reported accuracy of FoldX is 0.46 kcal/mol (i.e., the SD of the difference between ΔΔGs calculated by FoldX and the experimental values. The ΔΔG values are classified into seven categories regarding their impact on protein stability (Supplementary Table S1).

### Protein stability of human VRK1 pathogenic variants identified in patients

**Figure 2:**
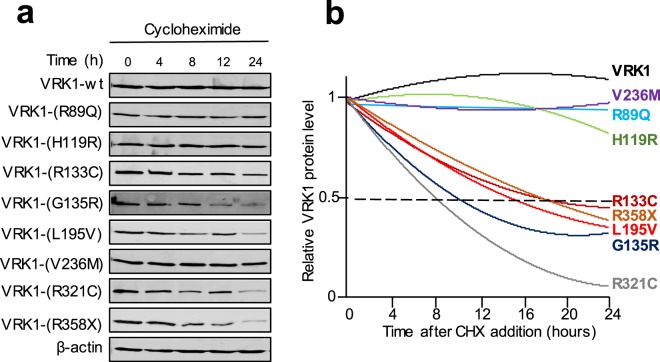
Stability of the proteins containing aminoacid changes in the VRK1 protein and detected in neuromotor syndromes associated to *VRK1* mutations. Each plasmid construct expressing a VRK1 variant protein was transfected in HEK 293T cells and cell lysates were prepared at different time points after cycloheximide (CHX) addition. (**a**) Western blot of each individual VRK1 pathogenic variant protein. The β-actin is representative. The individual β-actin for each variant are shown in Supplementary Fig. S7. (**b**) Stability of the VRK1 pathogenic variant proteins representing the mean of three independent experiments for each variant. Stability of the VRK1 mutant proteins follow a polynomial regression. The correlation between different time points and protein level for each individual variant and their fit (R values) by polynomic regression analysis^87^ are shown in Supplementary Fig. S7. For stable protein variants, their levels do not change with time and therefore there is no correlation.

The stability of the different VRK1 pathogenic variants was determined by transfection of *VRK1* variant constructs in HEK-293T cells and after addition of cycloheximide to block translation, the level of the pathogenic variant proteins was determined at different time points (Fig. 2). Two groups of variants were identified based on the stability of the protein. The stability was statistically analyzed by determining the fit by polynomic regression to detect the correlation between two variables, protein level and time. There VRK1 variants, V236M, R89Q and H119R form a group whose stability is similar to the wild type VRK1 (Fig. 2a,b), therefore the protein level does not correlate with the time. Another group includes VRK1 variants R133C, R358X, L195V, G135R and R321C, which are much more unstable and have a significantly shorter half-life (Fig. 2a,b). In these unstable variants, there is a very good correlation between the two variables of each variant, protein level as a function of time (individual data points are shown in Supplementary Fig. S7). For stable variant proteins, there is no correlation because their protein level remains constant throughout time.

### Kinase activity and specificity of VRK1 pathogenic variants

To identify whether there are differences in the kinase activity of VRK1 pathogenic variants, two types of assays were performed. One is autophosphorylation of the kinase^32,33^, and the other one is the phosphorylation of known specific residues in proteins that participate in different functions regulated by VRK1. All proteins in the assay, kinase and substrates, were equimolar at the low micromolar range (indicated in the methods section), and the rate limiting step of the assay is the concentration of ATP (5 µM), which is fifteen times below the VRK1 Km for ATP^32^. These permitted to perform the assays in the linear range of the activity.

**Figure 3:**
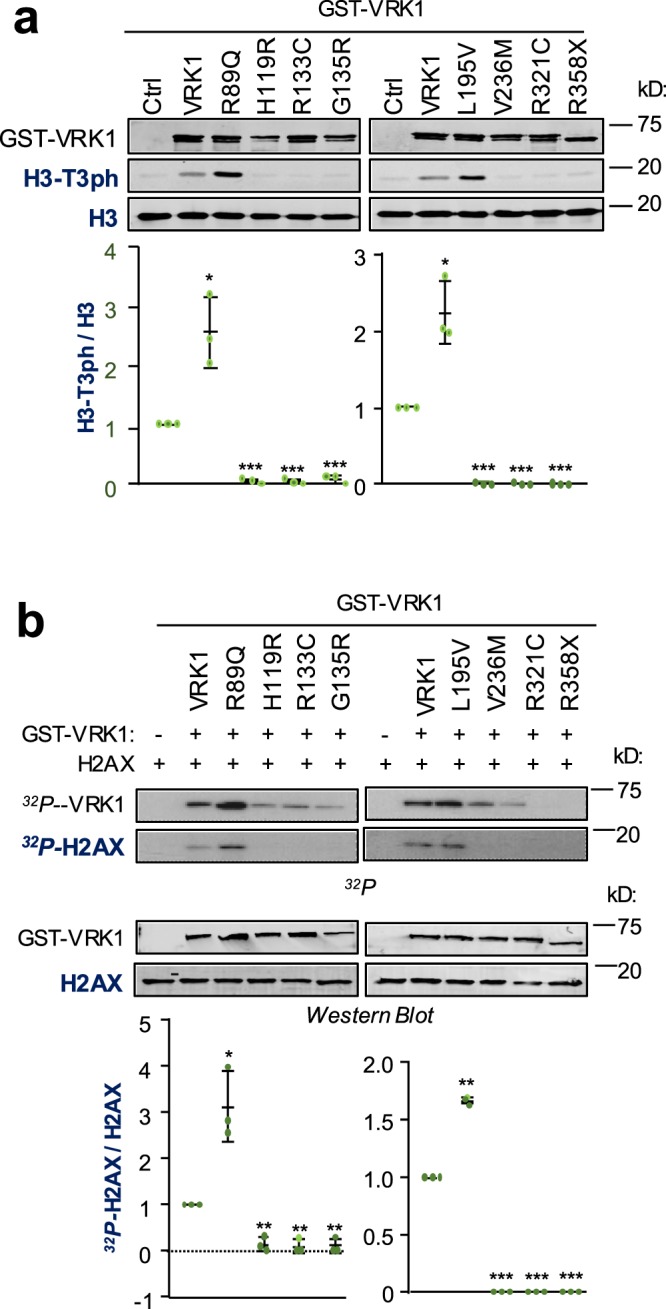
Effect of human VRK1 pathogenic variants on chromatin substrates: histones H3, forming part of nucleosomes, and H2AX associated to the initial reaction to DNA damage. (**a**) Phosphorylation of histone H3 in Thr3 by different VRK1 variant proteins. The H3T3 phosphorylation was detected in immunoblots with a phosphospecific antibody. (**b**) Phosphorylation of histone H2AX by different VRK1 pathogenic variant proteins performed by *in vitro* radioactive kinase assay. The graph shows the ratio of phosphorylated and non-phosphorylated substrates for each protein. The statistical study of the variables, protein level and time is a covariance analysis by least-square non-linear regression to detect the correlation between the change in protein as a function of time. The assays were performed in triplicate and are represented individually as dots in the graph. Wild type VRK1 was the reference value. *p < 0.05, **p < 0.005, ***p < 0.0005. Western blots with the proteins used in kinase assays are shown in Supplementary Fig. S7.

In the context of chromatin, the phosphorylation of two histones were studied. Histone H3 is a component of normal nucleosomes, which are remodeled based on the function of chromatin. The phosphorylation of Histone H3 in Thr3 was determined with a specific monoclonal antibody^3^. Most VRK1 variants have lost their ability to phosphorylate H3, with the exceptions or R89Q and L195V that have an increased activity (Fig. 3a).

Histone H2AX is associated to early stages in DNA damage responses^2^. The phosphorylation was determined by *in vitro* kinase with labeled ATP. The pattern of phosphorylation also detected a lost activity with all variants, except for R89Q and L195V that are more active (Fig. 3b). However, this reduction in activity was less noticeable regarding the autophosphorylation of VRK1, but have the same pattern.

**Figure 4:**
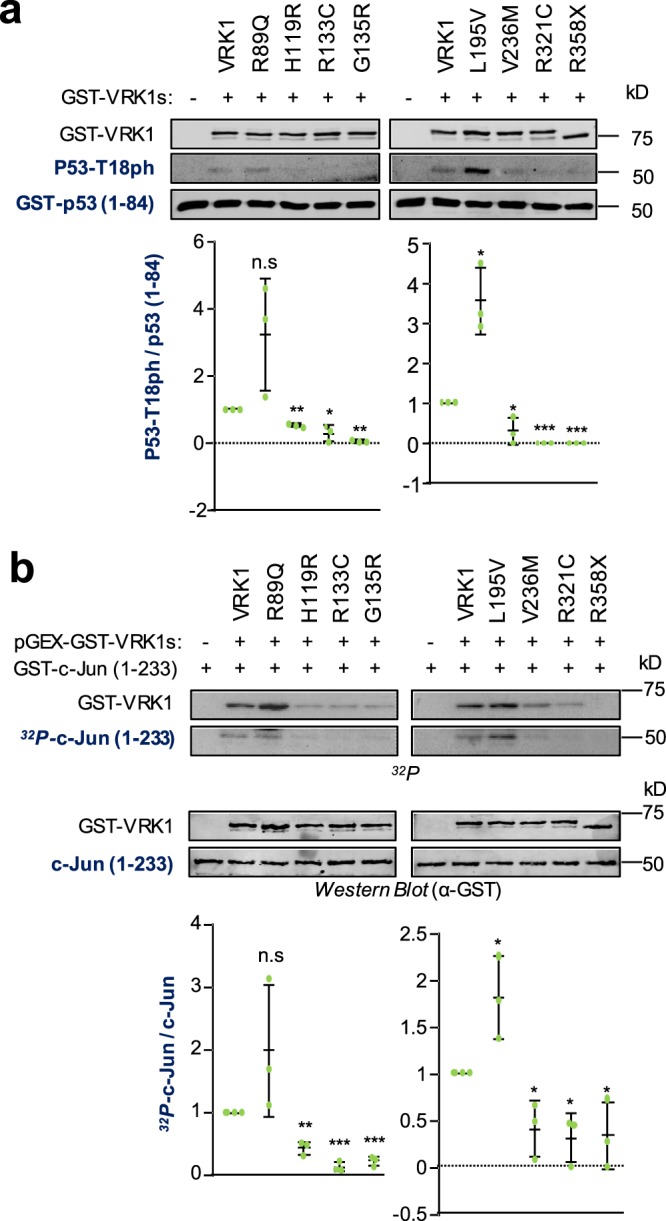
Effect on the kinase activity of VRK1 pathogenic variants that target transcription factors involved in responses to cellular stress. (**a**) VRK1 phosphorylation of in p53-Thr18 within its transactivation domain. As substrate p53 (1–84) transactivation domain. The specific phosphorylation in Thr-18 was detected with a specific monoclonal antibody. (**b**) VRK1 autophosphorylation and phosphorylation of c-Jun. As substrate, a construct of GST fused to the transactivation domain of c-Jun (1–223). The phosphorylation was performed in an *in vitro* kinase assay with radiolabeled ATP. The graph shows the ratio of phosphorylated and non-phosphorylated substrates. The statistical analysis is two-sided t-test with Welch’ correction. The assays were performed in triplicate and are represented individually as dots in the graph. Wild type VRK1 was the reference value. *p < 0.05, **p < 0.005, ***p < 0.0005.

A second group of proteins phosphorylated by VRK1 is formed by transcription factors that are implicated in DNA damage or cellular stress responses. In this context, the effect of VRK1 pathogenic variants on p53 and c-Jun was determined. VRK1 phosphorylates p53 in Thr18^5,6,34^. The phosphorylation of p53 by VRK1 pathogenic variants was determined in a kinase assay using as substrate the transactivation domain of p53 fused to GST (1–84)^5^. The phosphorylation was detected with a p53T18ph phospho specific antibody^6,35^. Only the R89Q and L195V VRK1 pathogenic variants were able to specifically phosphorylate p53, also at higher levels than the wild-type (Fig. 4a). Next, the effect of the VRK1 variants was tested on c-Jun, a transcription factor involved in cellular stress responses^36^. The phosphorylation of c-Jun regulatory domain was tested with a GST-C-Jun (1–233) fusion protein as substrate in a radioactive kinase assay. Only R89Q and L195V pathogenic variants were able to phosphorylate c-Jun regulatory region at higher levels (Fig. 4b).

**Figure 5:**
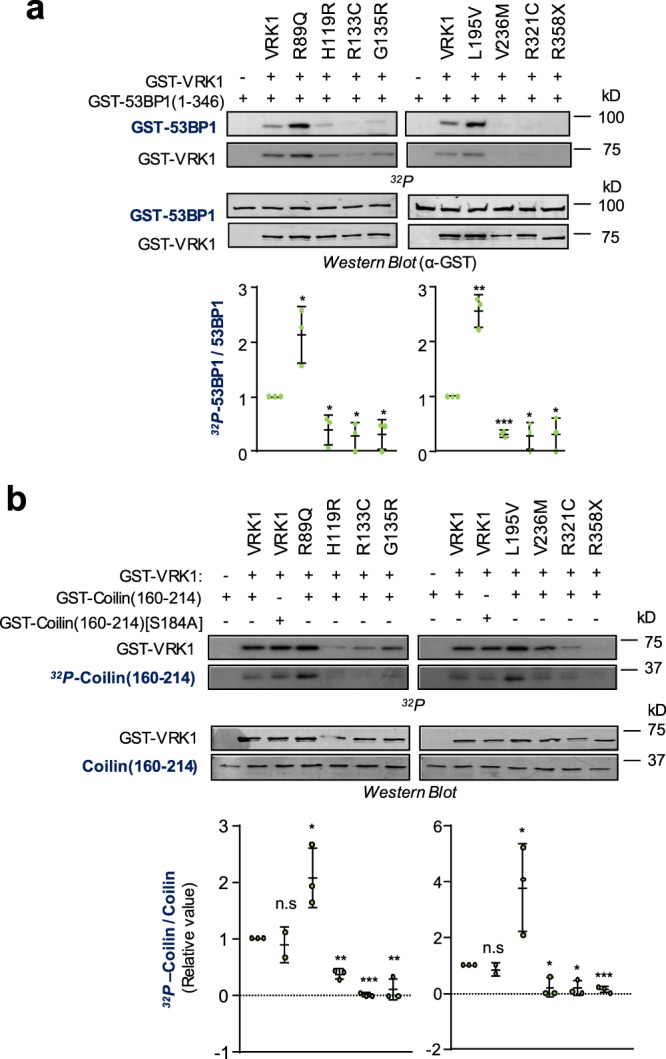
Effect of VRK1 pathogenic variants on the phosphorylation of 53BP1implicated in DNA repair by NHEJ, and phosphorylation of coilin implicated in the assembly of Cajal bodies. (**a**) VRK1 autophosphorylation and phosphorylation of 53BP1. As substrate, a construct of GST fused to the N-terminal domain of 53BP1 (1–346) was used. The phosphorylation was performed in an *in vitro* kinase assay. (**b**) VRK1 autophosphorylation and phosphorylation of coilin. As substrate, a construct of GST fused to the coilin domain (160–264) was used. The *in vitro* kinase assay was performed with radiolabeled ATP. The graph shows the ratio of phosphorylated and non-phosphorylated substrates. The statistical analysis is two-sided t-test with Welch’ correction. The assays were performed in triplicate and are represented individually as dots in the graph. Wild type VRK1 was the reference value. *p < 0.05, **p < 0.005, ***p < 0.0005.

The third group of substrates tested is formed by proteins associated to specific processes in which VRK1 participates, such DNA damage responses and Cajal bodies assembly. VRK1 regulates DNA damage response that is repaired by the Non-homologous end-joining route (NHEJ), where 53BP1 is a key protein that is known to be phosphorylated by VRK1^12,37^. As substrate it was used a fusion protein GST-53BP1 protein in the radioactive kinase assay. Only R89Q and L195V variants were able to phosphorylate 53BP1, also at higher levels than the wild type (Fig. 5a).

Coilin is a scaffold protein that is necessary for the organization of Cajal bodies (CB), which are dynamically assembled and disassembled during mitosis^38^, and regulated by VRK1^19^. Coilin form complexes with ataxin-1/SCA1^39^ and SMN^40^, two proteins related to some symptoms of the clinical phenotypes. Coilin is known to be phosphorylated in several residues by VRK1^19,23^. Therefore, its phosphorylation by VRK1 was also determined (Fig. 5b). In this case, also R89Q and L195V were able to phosphorylate coilin more than double than the kinase wild type.

### The phosphorylation effect depends on VRK1 pathogenic variants and not on the substrates

**Figure 6:**
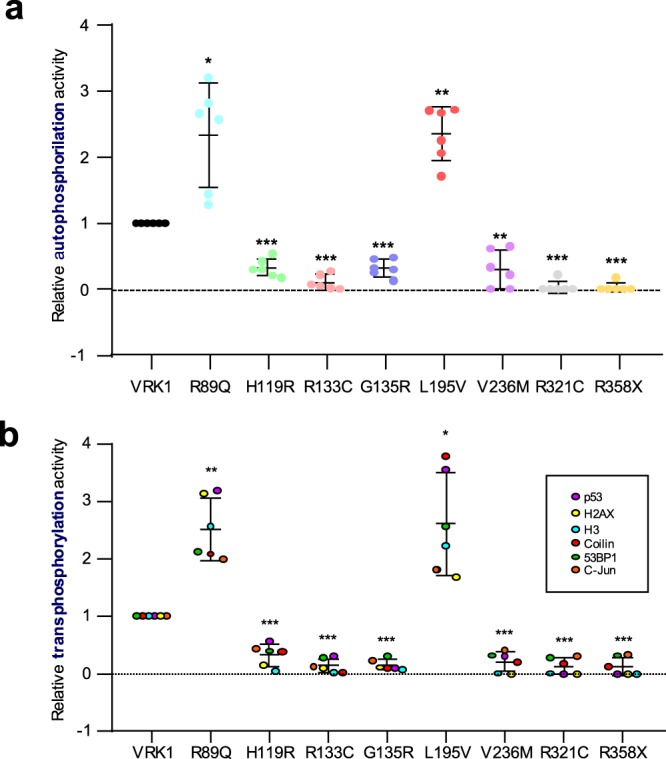
Autophosphorylation and transphosphorylation kinase assay of VRK1 pathogenic variants. (**a**) Quantification of the autophosphorylation of VRK1 using results from the previous radioactive kinase assays in which other substrates were included (images shown in Figs 3, 4 and 5). The points represent six independent kinase assays. The statistical analysis is two-sided t-test with Welch’ correction and are represented individually as dots in the graph. Wild type VRK1 was the reference value. *p < 0.05, **p < 0.005, ***p < 0.0005. (**b**) Transphosphorylation of the VRK1 substrates by determination of the ratio phosphorylated /non-phosphorylated substrate, independently of the type of substrates. Statistics on normalized combined data from assays using H2AX, c-Jun, 53BP1 and coilin as phosphorylation substrates. The points represent six independent kinase assays. The statistical analysis is two-sided t-test with Welch’ correction and are represented individually as dots in the graph. Wild type VRK1 was the reference value. *p < 0.05, **p < 0.005, ***p < 0.0005.

The differences in kinase activity might be a property either of the VRK1 pathogenic variants or of the specific phosphorylated substrate. To address this issue we reanalyzed the data from the previous autophosphorylation and trans-phosphorylation assays (Figs 3, 4 and 5). The wild-type VRK1 protein is autophosphorylated in multiple residues^5^. Therefore, the use of the radioactive kinase assay also permitted to determine the effect of VRK1 pathogenic variants on its autophosphorylation activity. This autophosphorylation assay was performed in the previous experiments for H2AX, c-Jun, 53BP1 and coilin. The results regarding VRK1 autophosphorylation and its quantification are shown in Fig. 6a. Both, the R89Q and L195V pathogenic variants are more active, and the rest of the VRK1 variants have a significant reduction in their kinase activity. To rule out that differences may be due to the substrates used, we reanalyzed the transphosphorylation data independently of the substrate based on the combined data from the six different substrates (Fig. 6b). The VRK1 pathogenic variants have a similar activity in both autophosphorylation and transphosphorylation reactions, suggesting that the alteration in VRK1 activity does not affect its specifity regarding the substrates used.

### Cajal bodies are reduced by the G135R mutation

**Figure 7:**
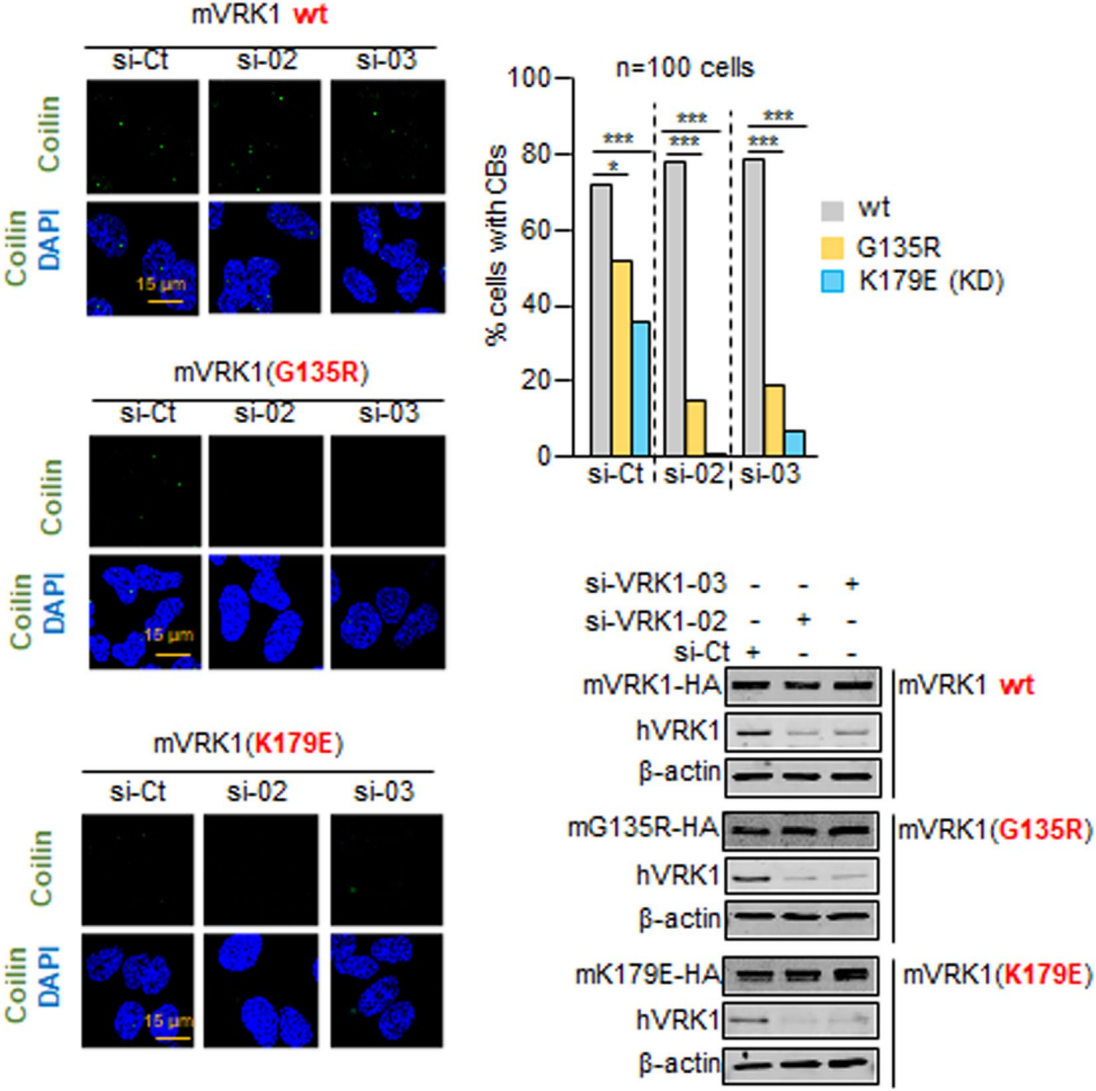
Effect of the mVRK1(G135R) variant on Cajal bodies. The mVRK1 wild type (wt) and the kinase-dead mVRK1(K179E) were used as positive and negative controls. The graph shows the number of cells with and without Cajal bodies. *p < 0.05, **p < 0.005, ***p < 0.0005. At the bottom is shown a western blot with the levels of human VRK1 and murine VRK1 wild type, mVRK1-(G135R) and kinase-dead mVRK1(K179E).

Coilin is the scaffold protein of Cajal bodies (CB) and is required for their assembly^41,42^. Coilin forms stable complexes with three neuropathological proteins: SMN, the spinal muscular atrophy protein^43^, ataxin-1 (SCA1), associated to spinocerebellar ataxia^39^, and VCP that is associated to some rare forms of amyotrophic lateral sclerosis^44,45^. Coilin is substrate of VRK1^23^ that regulates its stability and assembly in CB^19^. It is known that inactivation of the VRK1 catalytic site alters the formation of Cajal bodies^19^. Therefore, we determined whether the G135V variant could interfere with formation of Cajal bodies assembled on coilin in cell lines expressing this variant in the murine VRK1 gene. This variant was introduced in the murine *VRK1* gene (mVRK1), which was expressed by a lentiviral construct used to make stable cell lines. The human endogenous VRK1 was depleted by two different human siVRK1, and the effect on Cajal bodies of the murine mVRK1-G135R variant, or the kinase-dead mVRK1-K179E as control^19^, were determined. Both the mVRK1-G135R variant and the kinase-dead mVRK1-K179E mutant resulted in defective formation of Cajal bodies (Fig. 7).

### Formation of 53BP1 foci induced by DNA damage are impaired by the G135R variant

**Figure 8:**
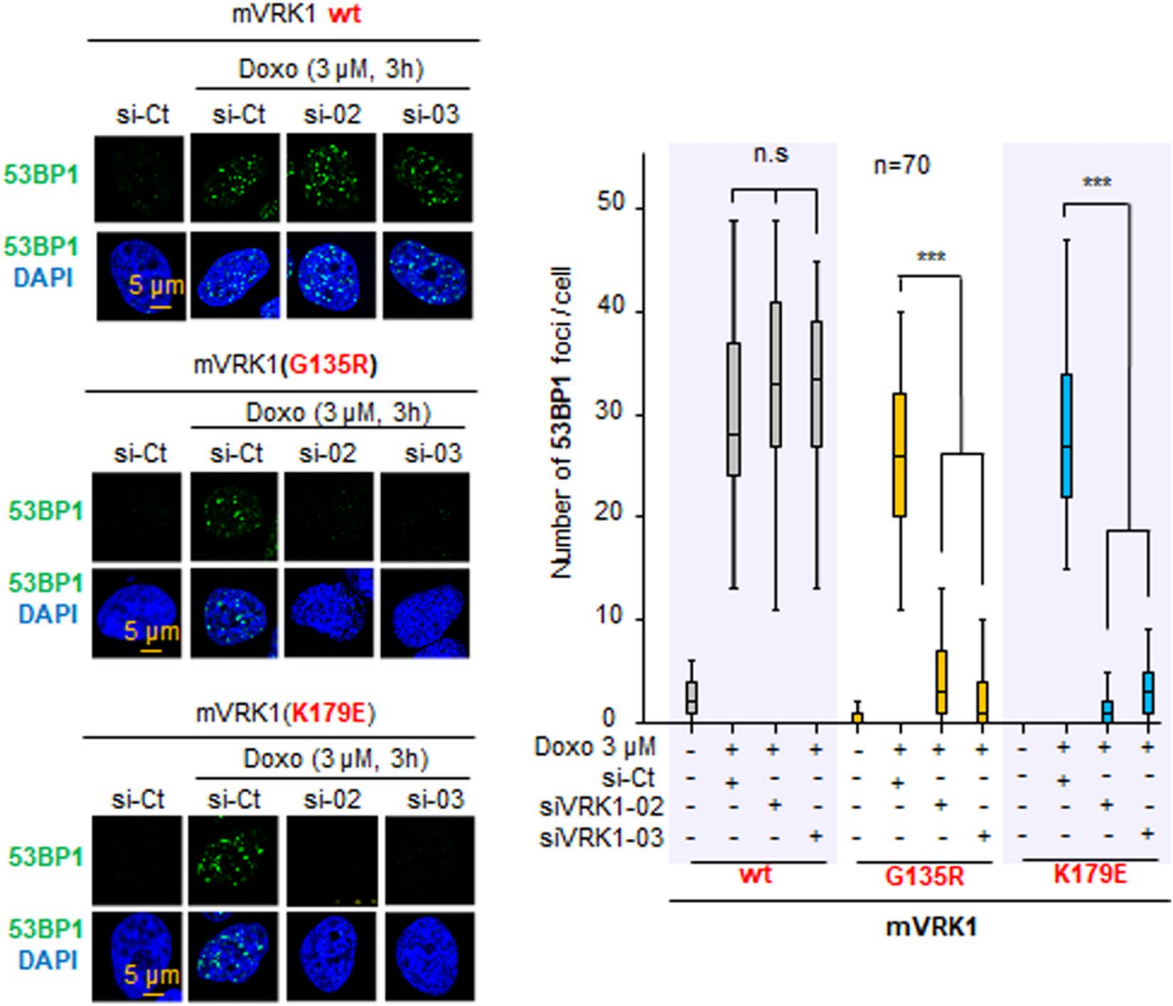
Effect of the mVRK1(G135R) variant on 53BP1 foci induced by DNA damage. The mVRK1 wild type and the kinase-dead mVRK1(K179E) were used as positive and negative controls. Field image is shown in Supplementary Fig. S9. Hela cells expressing the murine VRK1 (mVRK1) wt (top), G135R (center) and kinase-dead (K179E) (bottom) were depleted of endogenous human VRK1 with two different siRNA followed by treatment with doxorubicin to induce 53BP1 foci. The quantification of the effect is shown in the graph to the right. *p < 0.05, **p < 0.005, ***p < 0.0005. The expression of the proteins detected in western blot is shown in Fig. 7.

To identify a cellular effect of the human mutations, we selected the G135R variant detected in distal muscular atrophy^46^. VRK1 is a protein involved in different aspects of DNA damage responses^2,11,12^. Therefore it was tested the effect of the response to DNA damage induced by doxorubicin by the mVRK1-G135R and the wild-type and the kinase-dead mVRK1- K179E, as controls, following depletion of the endogenous human VRK1, and treatment with doxorubicin. As marker of the DNA damage response, the formation 53BP1 foci was determined^47^. The G135R pathogenic variant and the kinase-dead K179E protein caused a loss of 53BP1 foci in response to DNA damage (Fig. 8).

## Discussion

The implication of VRK1 in the pathogenesis of severe neuromotor developmental processes is likely to be due to alterations of cellular processes it regulates. The underlying common functional consequences of VRK1 mutations in neurodevelopmental syndrome is an impairment of neuronal functions regulated by VRK1. The common observation in either homozygous or compound heterozygous alleles indicates that the combination of two *VRK1* rare pathogenic alleles leads to a functional insufficiency of the processes regulated by VRK1. Among these there are impairments in cell cycle progression^3,9,13,14,19,48^, in responses to DNA damage^11,12,37^, altered nuclear envelope assembly^20^, and also impaired neuronal differentiation by altered regulation of Sox2^48^. All these processes are critical during early neurodevelopment. Therefore, impairment of cell cycle progression can lead to microcephaly or hypoplasia^21^, which is observed in patients with VRK1 mutations^22,24,49^. The known functions of VRK1 in the context of cell cycle progression and proliferation^3,9,13,14,19,48^ are consistent with this role^49^. In a murine VRK1 gene-trap model with a residual level of VRK1 protein^50^ there is a reduction of brain mass and a motor impairment^51^, consistent with the situation in human compound heterozygous alleles.

Functionally, the most characterized pathogenic variant is R358X, which lacks its C-terminus. The C-terminal region of VRK1 is necessary for the kinase activity^23,30^ because its correct folding is necessary for kinase activation^30^. Therefore truncating variants in the regulatory C-terminal region area likely to lack kinase activity, as is the case for the R358X variant that truncates the protein^23^. Furthermore, the mRNA transcript coding for the variant VRK1 (R358X) is very unstable, and its level is not detectable in cells^22^. The regulatory role of this C-terminus is supported by its regulation by autophosphorylation in Thr355 and Thr390^32^, which is inducible by DNA damage^12^, Thr355 can also be phosphorylated by PKCδ linking its regulation to other signaling pathways that induce cell death^52^.

In other cases, the variant residues directly or indirectly affects the catalytic site. The R133 residue is important for catalysis by interacting with the adenosine moiety of ATP^30^, thus this R133C variant is likely to be catalytically inactive^26^. Gly135 interacts with ATP and is required for the kinase activity^30^, and the variant G135R has lost most of its activity, and is also unstable. These two residues, R133 and G135 undergo chemical shift perturbations and affect the DRF motif^53^. It is known that the loss of VRK1 kinase activity by itself makes this protein unstable^13^. The L195 is required for the interaction of the DYG motif with ATP^30^, thus the variant L195V might facilitate the activity of this variant due to the smaller size of valine. Two of the variants, R89Q and L195V have a higher activity in both autophosphorylation and phosphorylation of specific substrates such as H3 (Fig. 3a) and p53 (Fig. 3b). The L195V variant has a reduced half-life that might mimic a deficiency of the active kinase.

The contribution of VRK1 to neuropathogenesis can be a consequence of a defective organization of Cajal bodies (CB) by alteration of the regulation of coilin by VRK1, which is required for CB assembly and disassembly^19^. Coilin is associated to proteins such as SMN^43,54^, scaRNPs^54^ or ataxin-1^39^ that are implicated in severe neurological phenotypes such as muscular atrophy^55,56^ and ataxias^39^. Ataxin-1 (SCA1) is a phosphorylated protein^57,58^, and in this context, VRK1 mutations might be a pathogenic alternative to expansion of poly-Q expansion in ataxin-1^59^. Coilin also interacts with VCP, a protein associated to amyotrophic lateral sclerosis^44,60^. Therefore, an alteration in the organization and regulation of these coilin and Cajal body complexes by VRK1 variants is likely to have important neuropathological consequences, particularly since VRK1 regulates the proteasomal degradation of coilin^19^, which form complexes with several proteins associated to several neuromotor syndromes. Moreover, Cajal bodies are RNP-complexes that are altered in spinal muscular atrophy (SMA)^61^, and RNA binding proteins also play a not well known role in amyotrophic lateral sclerosis (ALS)^62^. Therefore, this function might be defective in patients with VRK1 pathogenic variants. In this context, the defective formation of Cajal bodies by the G135R variant (Fig. 7) is consistent with this interpretation.

VRK1 might also contribute to neurological syndromes as a consequence of its roles on neural cell motility. A reduction in VRK1 protein levels, independent of its activity, has been associated to a downregulation of amyloid-beta precursor protein (APP) that leads to a reduction in neuronal migration, which can be rescued by overexpression of APP^49^. Moreover, an effect of VRK1 on cellular motility has also been identified as contributing to breast cancer metastasis by facilitating mesenchymal to epithelial transition^63^. Therefore, defects in neuronal migration are likely to have important consequences in size and organization of the nervous system, leading to hypoplasia or microcephaly, and consequently cause severe neuromotor and neurodevelopmental delay syndromes.

An additional pathogenic role of VRK1 variants is likely to be a consequence of its roles in chromatin reorganization^1,16^. Alterations in histone posttranslational epigenetic modifications are involved in neurodegerative disorders by not yet known mechanisms^64,65^. Another alternative mechanism might be a consequence of the role that VRK1 plays in the cellular response to DNA damage^11,12,16,37,47^. It is well known that mutations in genes coding for proteins that participate in DNA-damage responses cause neurodegerative syndromes^66,67^, such as Nijmegen^68^, Cockayne syndromes^69^ and many others^67^, including amyotrophic lateral sclerosis^70^. Therefore, it is likely that alterations in DDR by a VRK1 deficiency can lead to neurodevelopmental phenotypes. There is an extensive neuropathology associated to defects in genes coding for proteins that participate in different aspects of DNA repair processes. Among them is NBS1^71,72^ that is regulated by p53^72^. P53^6,73^ and NBS1 are both phosphorylation targets of VRK1^11^. Hereditary *ATM* (ataxia-telangiectasia mutated) mutations are also associated to neurological phenotypes^74,75^, and in this context VRK1 is an upstream regulator of ATM-mediated responses to DNA damage^2,11,12^. In this context the defective formation of 53BP1 foci in response to DNA damage by the G135R variant (Fig. 8) is consistent with this interpretation.

Furthermore, an additional factor that can explain the loss of stability is a consequence of the lack of data on the protein interaction of VRK1 with its substrates, as well as its recognition by regulatory proteins and active degradation mechanisms. VRK1 can be degraded in the lysosome^35^, but this requires VRK1 nuclear export and lysosomal targeting. The variants might alter interactions with other regulatory or interacting proteins, but there is no available data on any of these VRK1 protein complexes. Another factor might be due to VRK1 effects that are independent of the kinase activity. Kinase-dead VRK1 has been shown to regulate neuronal migration in murine embryo development^49^.

The heterogeneity or variability among the clinical phenotypes associated to *VRK1* pathogenic variants are likely to be conditioned by additional genetic alterations present in the affected families, since all cases occurred either in consanguineous families or within a specific ethnic subgroup, which might have a higher frequency incidence of pathogenic variant alleles. In addition, genetic variations or alterations, such as copy number variations (CNVs) or pathogenic variants in other genes, which differ among patients, can significantly contribute to the heterogeneity of the clinical phenotypes identified. A genetic heterogeneity has already been reported in other complex neurological diseases, such as epilepsy^76,77^ and neurodevelopmental delays^78–80^.

In this report we conclude that the common underlying effect of human *VRK1* pathogenic variants identified in patients with neuromotor and neurodevelopmental syndromes is a functional insufficiency of VRK1 resulting from an altered protein stability or reduced kinase activity that impair cellular functions regulated by VRK1.

## Materials and Methods

### Molecular modelling of missense VRK1 mutations

Modelling of the human VRK1 pathogenic variant proteins was performed using as reference the published structures 2RSV^29^ and 2LAV^30,53^ available from the Protein Data Bank. FoldX program^81^ (http://foldxsuite.crg.eu/) was used to predict the effect of missense mutations in the structural stability of the VRK kinase (PDB identifier 2LAV). This structure was selected, as its partial C-terminal domain is correctly located within the structure as compared to 2RSV. For each protein variant we calculated ten iterations to ensure that the algorithm reaches convergence. The reported accuracy of FoldX is 0.46 kcal/mol (i.e., the SD of the difference between ΔΔGs calculated by FoldX and the experimental values). We can bin the ΔΔG values into seven categories of stability (Supplementary Fig. S1). Regarding the van der Waals clashes, this indicates whether the mutation impairs drastically the structure of the protein. We studied the following mutations, and represented the relative TE differences using an R custom script to generate the image files. The residue numbering corresponds to the PDB structure. 89:(R/Q), 119:(H/R), 133:(R/C), 135:(G/R), 195:(L/V), 236:(V/M), and 321:(R/C). Mutations and interactions are illustrated using Pymol (https://pymol.org/2/). All the figures represent the interaction of residues forming an interaction network via hydrogen bonds.

### Plasmids and mutagenesis

Human VRK1 was expressed from mammalian expression vector, pCEFL-HA-VRK1^82^, and bacterial expression pGEX-4T-VRK1^3,5,82^. These plasmids were used as targets to generate the pathogenic variants identified in patients. The primers used to generate the VRK1 mutations are listed in Supplementary Table S2. Mutations in VRK1 were performed using the Quickchange site-directed mutagenesis kit (Stratagene-Thermo-Fisher). Sanger sequencing was used to confirm all variants generated using the following primers VRK1-forward (5′-CCTCGTGTAAAAGCAGCTCAAGCTG-3′) and VRK1-reverse (5′-GGACTCTCTTTCTGGTTCTTGAACGG-3′). The kinase-dead VRK1, K179E mutation, has already been reported^13^. VRK1 wild type and variants (R89Q, H119R, R133C, G135R, L195V, V236M, R321C, R358X) were expressed from constructs pGEX4T-GST-VRK1 plasmid expressed in *E*. *coli* BL21 strain competent cells. The following plasmids were used to express the substrates: plasmid pGEX4T-GST-c-Jun (1–233)^7^, pGST4T-53BP1 (1–346) was a gift of J. Chen^12,83^; GST-p53 (1–85) was from D. Meek (1–85)^5,34,84^, and pGEX4T-GST-Coilin (160–214) [S184A]^19^.

All plasmids were expressed in BL21 *E*. *coli* to express and purify the fusion protein used as substrate in kinase assays. The purity of purified kinases and substrate proteins are shown in Supplementary Fig. S8.

### Cell lines, transfection and cell lysate

HEK-293T (ATCC-CRL-11268) and HeLa (ATCC-CCL2) validated cell lines were grown in Dulbecco’s Modified Eagle’s Medium (DMEM) (Sigma-Aldrich) supplemented with 10% Fetal Bovine Serum (FBS), 2 mM L-glutamine, and penicillin (50 U/ml)/streptomycin(50 µg/µl) (GIBCO-life technologies)^19,48^. Cells were transfected using Lipofectin as previously described^11,19,48^. The plasmid expressing the pathogenic variants were used in an amount chosen to express a similar initial level of protein, which was completed with empty vector plasmid to reach the same final concentration of DNA in the transfection.

Cell extract were prepared by using a mild lysis buffer (50 mM Tris-HCl, pH 8.0, 150 mM NaCl, 1% Triton X-100 and 1 mM EDTA) supplemented with protease inhibitors (1 mM PMSF, 10 µg/mL aprotinin and 10 µg/mL leupeptin) and phosphatase inhibitors (1 mM sodium orthovanadate, 1 mM NaF) and incubating for 20 minutes. Cell lysates were centrifuged for 20 minutes at 16.100 g to remove debris and supernatants were used for the study.

Murine VRK1, wild-type and kinase dead (K179E) were also made in a lentiviral expression construct, plasmids pLenti-C-HA-IRES-BSD-mVRK1 and pLenti-C-HA-IRES-BSD-mVRK(K179E). The human G135R mutation was introduced in the murine VRK1 plasmid pLenti-C-HA-IRES-BSD-mVRK1 to generate pLenti-C-HA-IRES-BSD-mVRK1(G135R). The variants were introduce with GeneArt Site-Directed Mutagenesis System (Invitrogen-ThermoFisher). The primers used were for G135R: (forward: 5′-GTTTATGATAATGGACCGCTTTCGGAGTGACCTTC-3′; reverse: 5′-GAAGGTCACTCCGAAAGCGGTCCATTATCATAAAC-3′) and for K179E (5′-GTGCACGGGGACATCGAGGCCTCCAACCTGCTCCT-3′; reverse:5′-CTCTGGTTTAGCAGCCTGCTGGTAGAACTTTAATTCCG-3′). The mutations were confirmed by DNA sequencing. These murine constructs were used to generate HeLa-derived stable cell lines expressing the variant protein and were selected with blasticidine. Several clones were obtained for each construct.

### Kinase assays

The kinase assays were performed as previously described^33,82^. Briefly, *In vitro* kinase assays with [^32^-P]-γATP were performed with GST-VRK1 wild-type and variants^19,48,82^. Assays with the following substrates were previously published: p53^34,85^, histone H3^2,3^, 53BP1^12^, ATF2^8^, c-Jun^7^, Sox2^48^. GST-coilin^19^. Recombinant and purified human histones H3^2,3^ and H2AX^2^ were from Merck/Millipore.

The Serine-Threonine kinase activity of VRK1 was analysed by performing *in vitro* kinase assays using 2 µg (3.7 µM) of GST-VRK1 and variant recombinant proteins that were purified from BL21 cells (Fig. S3). The following proteins (2 µg) were used as specific substrates, GST-53bp1(1–346) (1.5 µM)^12^, GST-Coilin(160–214) (3 µM), GST-Coilin(160–214)[A184] (3 µM)^19^, GST-p53(1–84) (2.7 µM)^5,32,86^, GST-C-Jun(1–233) (1.9 µM)^7^ or human recombinant histones H3 (6 µM)^2,3^ and H2AX (6 µM)^2^. To perform the kinase assay^32^, it was used a specific buffer for casein kinases (20 mM Tris-HCl pH 7.5, 5 mM MgCl_2_, 0.5 mM DTT and 150 mM KCl), 5 µM ATP and 5 µCi (0.1 µM) radiolabelled [ɣ-^32^P]ATP in a final volume of 40 µl^3,12,23,48^. The kinase assay was performed during 45 min at a temperature of 30 °C. This ATP concentration is fifteen times lower that the VRK1 Km for ATP^32^. Therefore, in the assay the kinase is working at a suboptimal low rate in its linear phase and permits long linear observation time. Some specific phosphorylation of proteins in the kinase assays were detected using antibodies for specific residues phosphorylated by VRK1. H3T3ph was detected with a rabbit polyclonal antibody (Upstate-Millipore)^2,48^. The p53T18ph in GST-p53(1–85) was detected with rabbit polyclonal (Abcam, Cambridge, UK)^34^. Film exposure was in the lineal response range for all assays.

### Electrophoresis, antibodies and immunoblots

The size-dependent separation of proteins was performed by vertical electrophoresis in SDS-PAGE gels under denaturing conditions in a running buffer (25 mM Tris-HCl, 200 mM glycine, 1.7 mM SDS). Proteins were transferred to a PVDF membrane (Immobilon-FL, Millipore) in transfer buffer (25 mM Tris-HCl, 19.2 ml glycine, 15% methanol) as previously described^11,19,37,48^. The primary and secondary antibodies used for this work are described in Supplementary Table S3. The secondary antibodies were incubated for an hour and the fluorescence was detected with LI-COR Odyssey Infrared Imaging System or with ECL Western Blotting Detection Reagent (SIGMA-ALDRICH) if the secondary antibodies were conjugated with peroxidase (Supplementary Table S3).

### Statistical analysis

Statistical analysis were performed using the IBM SPSS 28 statistics package. All assays were performed in the lineal response range and in identical conditions for all substrates. Individual quantitative experiments were repeated between three and six times, and statistical significance was analyzed using two-tailed T-test with Welch’ correction^87^. In all cases, the level of significance was: *p < 0.05; **p < 0.005; and ***p < 0.0005.

In stability experiments, data was analyzed using covariance least-square polynomic regression test to detect the correlation coefficient^87^.

### Reagents

Recombinant human histones H3 and H2AX (Millipore, Merck), Cycloheximide (Sigma-Aldrich). All other chemical were from Sigma-Merck (Darmstadt, Germany). Tissue culture media and reagents were from GIBCO-ThermoFisher Scientific (Waltham, MA).

## Supplementary information

Supplementary information

## Data Availability

All materials are available upon request. No datasets were generated in this study.
