# Supplementary material for: VRK1 functional insufficiency due to alterations in protein stability or kinase activity of human VRK1 pathogenic variants implicated in neuromotor syndromes
Source: Sci Rep. 2019 Sep 16;9:13381. doi: 10.1038/s41598-019-49821-7 (PMC6746721; doi:10.1038/s41598-019-49821-7)
Supplement: Supplementary file 1 — Supplementary information [file 41598_2019_49821_MOESM1_ESM.pdf]

Supplementary information

VRK1 functional insufficiency due to alterations in protein stability or kinase activity of human *VRK1* pathogenic variants implicated in neuromotor syndromes

Elena Martín-Doncel [1, 2], Ana M. Rojas [3, 4], Lara Cantarero [1, 2, 5] and Pedro A. Lazo [1, 2, *]

[1] *Experimental Therapeutics and Translational Oncology Program, Instituto de Biología Molecular y Celular del Cáncer, Consejo Superior de Investigaciones Científicas (CSIC) - Universidad de Salamanca, Salamanca, Spain*

[2] *Instituto de Investigación Biomédica de Salamanca (IBSAL), Hospital Universitario de Salamanca, Salamanca, Spain*

[3] *Centro Andaluz de Biología del Desarrollo, CSIC-Universidad Pablo de Olavide, Sevilla, Spain*

[4] *Instituto de Biomedicina de Sevilla (IBIS), CSIC-Universidad de Sevilla, Hospital Universitario Virgen del Rocío, Sevilla, Spain*

[5] *Laboratorio de Neurogenética y Medicina Molecular, Institut de Recerca Sant Joan de Déu, Esplugues de Llobregat, Barcelona, Spain.*

Supplementary Table S1.

Prediction of VRK1 pathogenic variant protein stability based on the variations in free energy values (ΔΔG) values. The variants can be distributed into seven categories. The reported accuracy of FoldX is 0.46 kcal/mol (i.e., the SD of the difference between ΔΔGs calculated by FoldX and the experimental values).

**Effect on Stability**	**ΔΔG**
High Stability	ΔΔG < −1.84 kcal/mol
Stabilizing	−1.84 kcal/mol ≤ ΔΔG < −0.92 kcal/mol
Light stability	−0.92 kcal/mol ≤ ΔΔG < −0.46 kcal/mol
neutral	−0.46 kcal/mol < ΔΔG ≤ +0.46 kcal/mol
Light destabilizing	+0.46 kcal/mol < ΔΔG ≤ +0.92 kcal/mol
Destabilizing	+0.92 kcal/mol < ΔΔG ≤ +1.84 kcal/mol
High destabilizing	ΔΔG > +1.84 kcal/mol

Supplementary Table S2.

Primers used for generating human *VRK1* pathogenic variants

Mutation	primer	Sequence
**R89Q**	R89Q-*Forward*	5'-AATTAAAGTTCTACCAACAAGCTGCAAAACCAGAGCA-3'
	R89Q *Reverse*	5'-TGCTCTGGTTTTGCAGCTTGTTGGTAGAACTTTAATT-3'
**H119R**	H119R *Forward*	5'-TCTGGTCTACGTGACAAAAATGGAA-3'
	H119R *Reverse*	5'-TTCCATTTTTGTCACGTAGACCAGA-3'
**R133C**	R133C *Forward*	5'-GGTTTATGATAATGGATTGCTTTGGGAGTGACCTTCAG-3'
	R133C *Reverse*	5-CTGAAGGTCACTCCCAAAGCAATCCATTATCATAAACC-3'
**G135R**	G135R *Forward*	5'-GGTTTATGATAATGGATCGCTTTAGGAGTGACCTTCAG-3'
	G135R *Reverse*	5'-CTGAAGGTCACTCCTAAAGCGATCCATTATCATAAACC-3'
**L195V**	L195V *Forward*	5'-GGCCTCAAATCTTCTTGTGAACTACAAGAATCCTGACC-3'
	L195V *Reverse*	5'-GGTCAGGATTCTTGTAGTTCACAAGAAGATTTGAGGCC-3'
**V236M**	V236M *Forward*	5'-ATCGACGCTCACAAAGGCATGGCCCCATCAAGACG-3'
	V236M *Reverse*	5'-CGTCTTGATGGGGCCATGCCTTTGTGAGCGTCGAT-3'
**R321C**	R321C *Forward*	5'-TGAAAATTTATGTGACATTCTTTTGCA-3'
	R321C *Reverse*	5'-TGCAAAAGAATGTCACATAAATTTTCA-3'
**R358X**	R358X *Forward*	5'-GACCAGCCTCAAAGAAGTAGAAGAAAGAAGCAGAAGAAAGC-3'
	R358R *Reverse*	5'-GCTTTCTTCTGCTTCTTTCTTCTACTTCTTTGAGGCTGGTC-3'
**K179E**	K179E *Forward*	5'-AGTATGTGCATGGAGATATCGAGGCCTCAAATCTTCTTCTGAACT-3'
	K179E *Reverse*	5'-AGTTCAGAAGAAGATTTGSGGCCTCGATATCTCCATGCACATACT-3'

Supplementary Table S3.

List of primary and secondary antibodies used in this work.

Antibody	Type	Dilution WB	Clone and/or reference	Supplier
GST-Tag	Mouse monoclonal	1:1000	B14/sc-138	Santa Cruz
HA-Tag	Mouse monoclonal	1:1000	F7/sc-7392	Santa Cruz
Histone H3	Rabbit polyclonal	1:1000	9175	Cell Signaling
Phospho-histone H3 (Thr3ph)	Rabbit polyclonal	1:1000	05-746R	Merck-Millipore
Phospho-p53 (Thr18ph)	Rabbit polyclonal	1:1000	ab30659	Abcam
β-actin	Mouse monoclonal	1:1000	AC15/A5441	Sigma-Aldrich
Goat Anti-Mouse IgG, DyLight 680	Goat	1:10000	35518	Thermo Scientific
Goat Anti-Rabbit IgG, DyLight 800	Goat	1:10000	35571	Thermo Scientific
Anti-Mouse IgG-Horseradish Peroxidase-Linked Species-Specific Whole Antibody	Sheep	1:10000	NA931V	GE-Amersham Biosciences

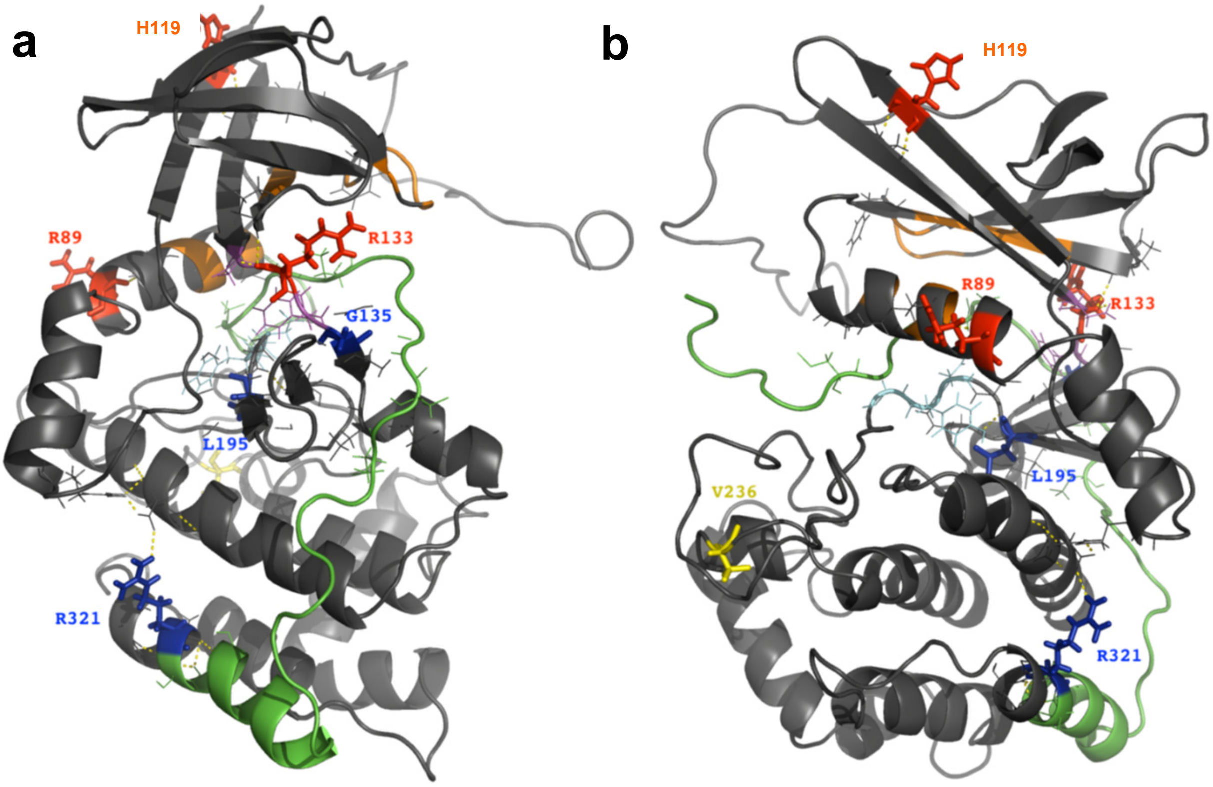

Supplementary Figure S1. Heterogeneous localization of the variant aminoacids on the three dimensional structure of human VRK1. Two different views by rotation of the structure (**a, b**) showing the location of VRK1 wild type aminoacids that are replaced by different variants associated to neuromotor developmental syndromes.

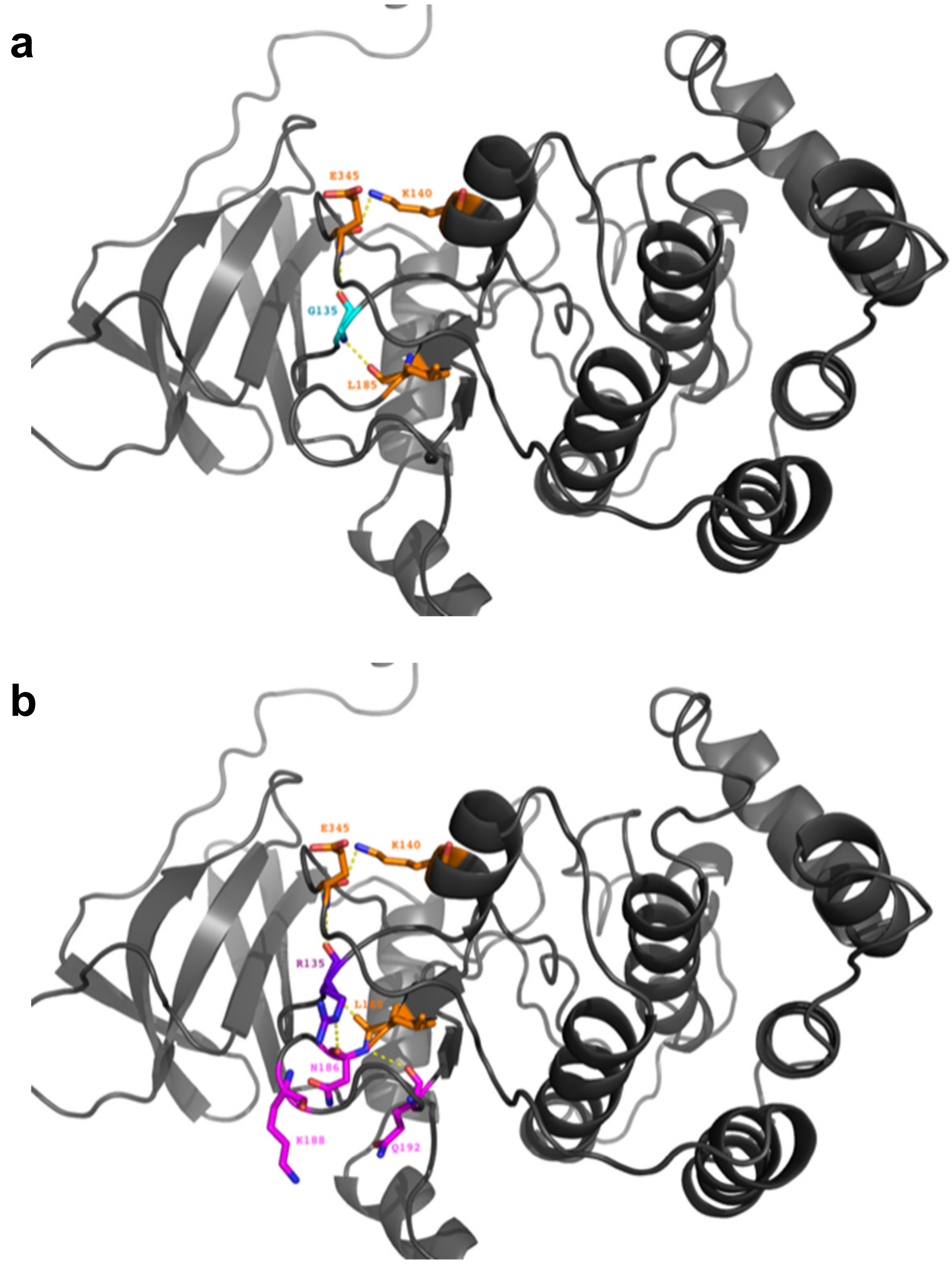

Supplementary Figure S2. Interaction networks of the wild type G135 (**a**) **and its pathogenic variant R135** (**b**). This variant has a destabilizing effect. When polar interactions are present within the interacting residues, they are indicated by yellow lines. In the graphics, the protein is represented as a gray cartoon. The interacting residues are orange, the WT residue is cyan, the mutated residue is purple.

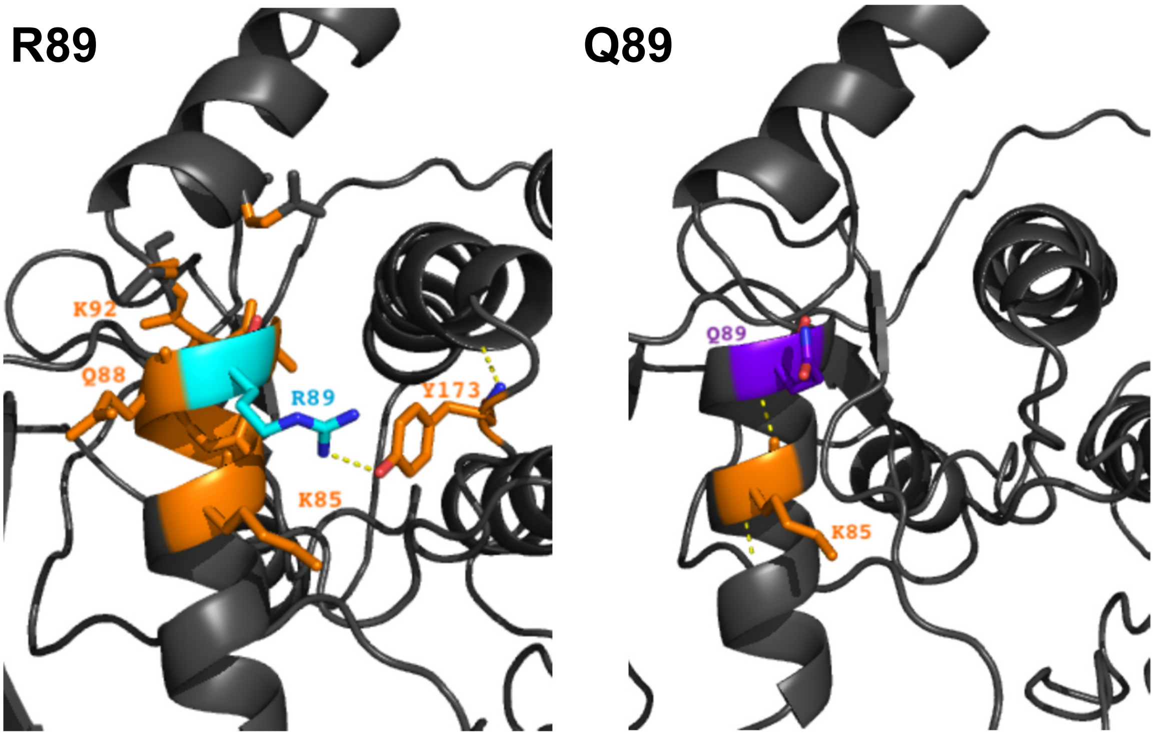

Supplementary Figure S3. Effect of the R89Q pathogenic variant. The WT residue is cyan, the mutated residue is purple. The interaction of R89 with Y173 is lost in the Q89 variant. When polar interactions are present within the interacting residues, they are indicated by yellow lines. In the graphics, the protein is represented as a gray cartoon. The interacting residues are orange, and when there are extended residues to the network that are represented in cyan..

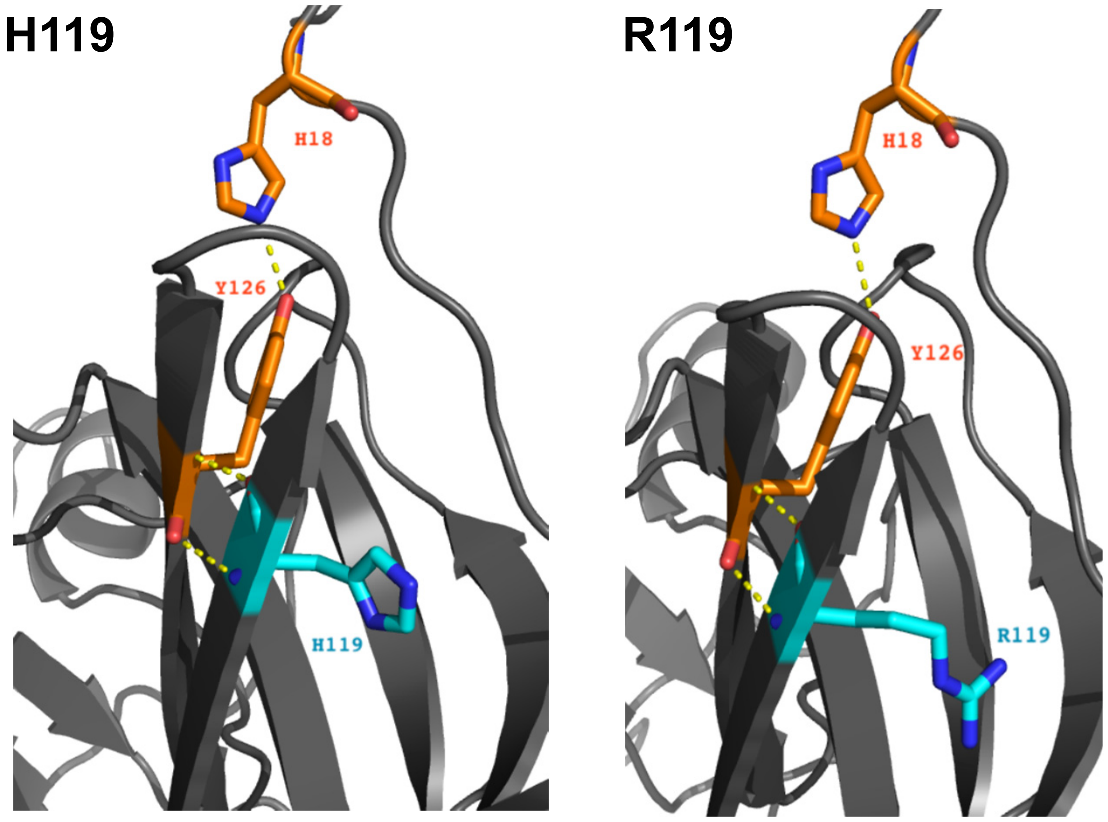

Supplementary Figure S4. Effect of the H119R pathogenic variant. When polar interactions are present within the interacting residues, they are indicated by yellow lines. In the graphics, the protein is represented as a gray cartoon. The WT residue is cyan, the mutated residue is purple. The interacting residues are orange, and when there are extended residues to the network that are represented in cyan. When polar interactions are present within the interacting residues, yellow lines indicate them.

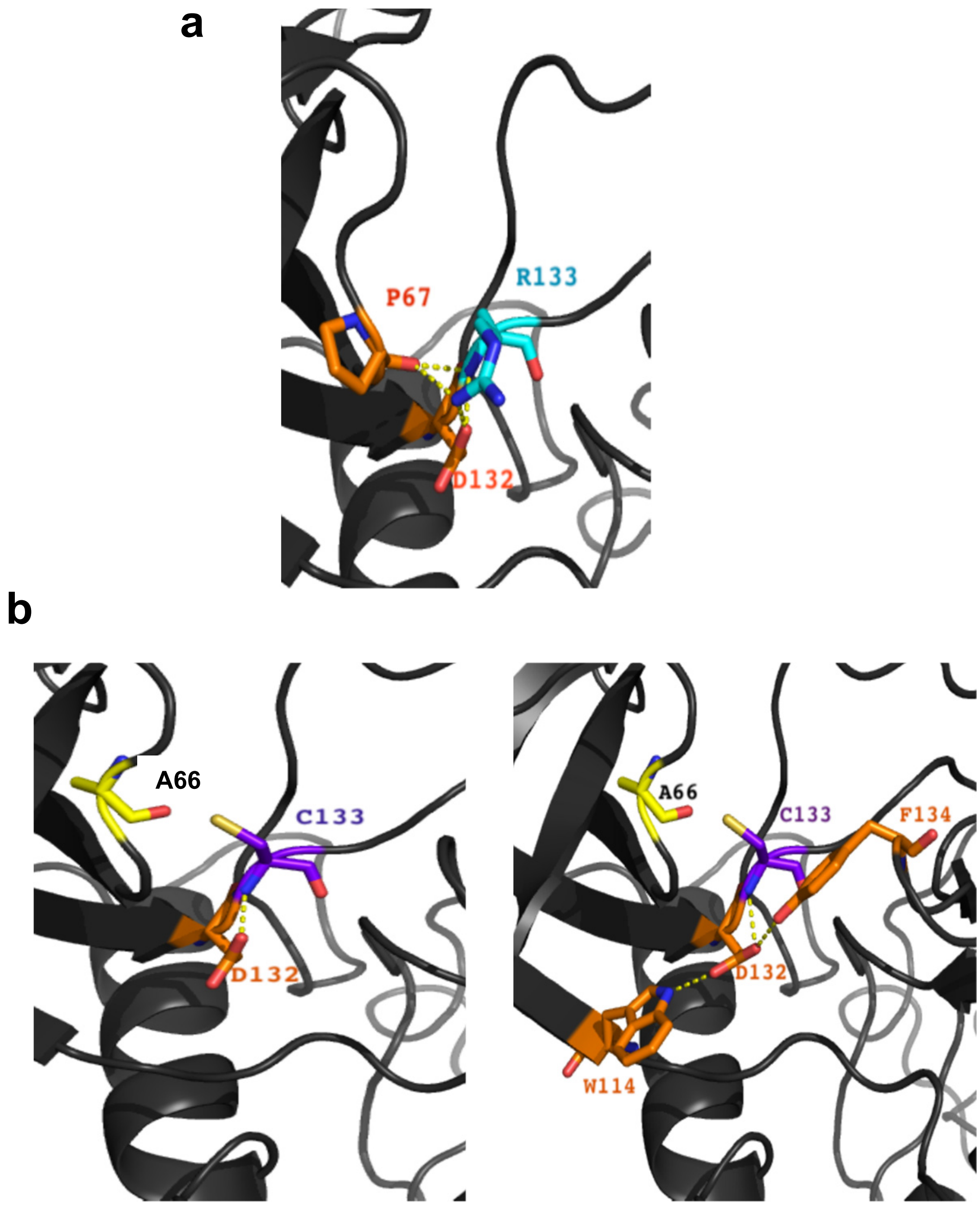

Supplementary Figure S5. Effect of the R133C pathogenic variant. **a**. Wild type R133. **b.** The C133 variant has two rotamers (left and right). When polar interactions are present within the interacting residues, they are indicated by yellow lines. In the graphics, the protein is represented as a gray cartoon. The WT residue is cyan, the mutated residue is purple. The interacting residues are orange.

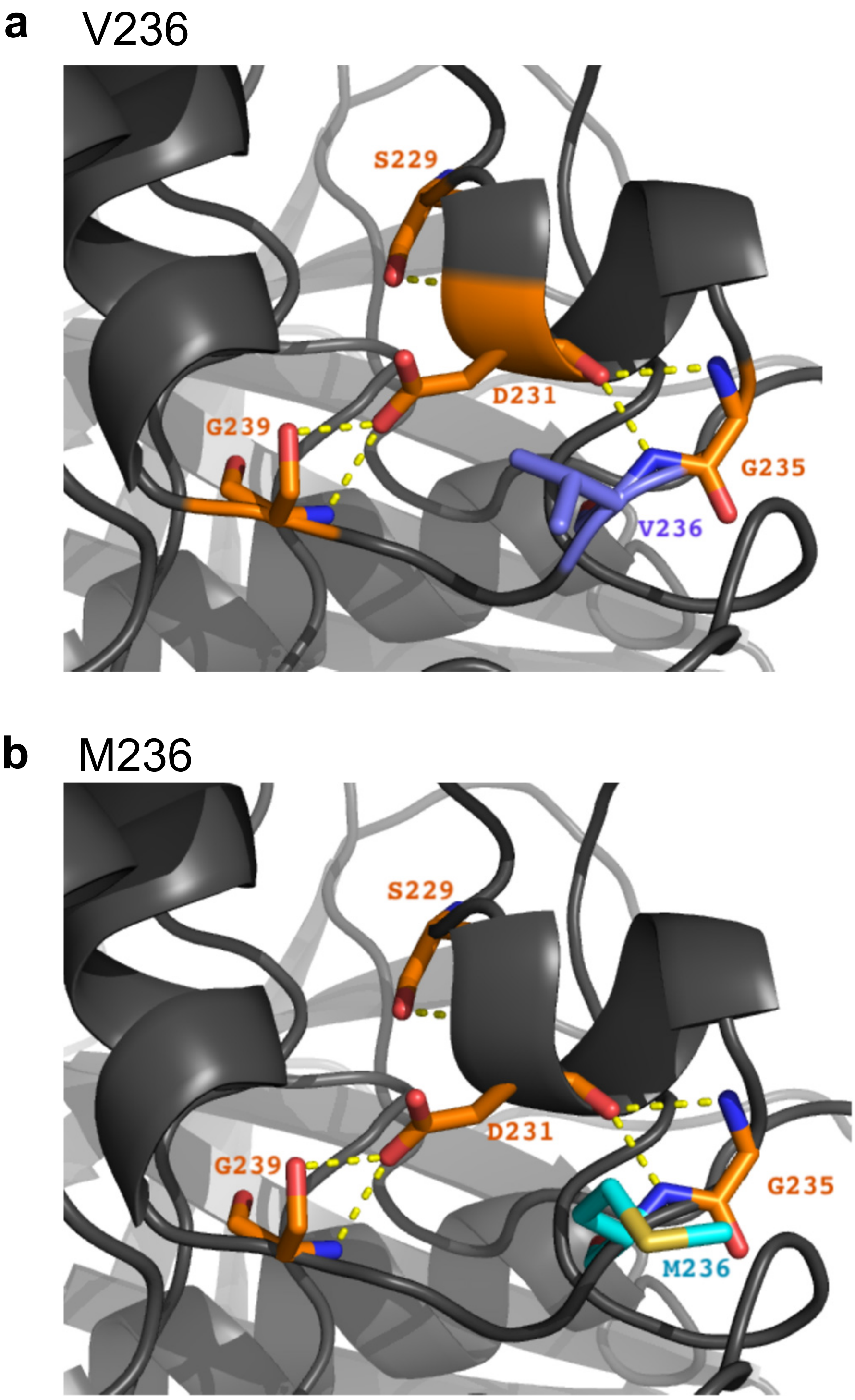

Supplementary Figure S6. Effect of the V236M pathogenic variant. The WT residue is cyan, the mutated residue is purple. When polar interactions are present within the interacting residues, yellow lines indicate them. The interacting residues are orange, and when there are extended residues to the network, these are represented in cyan.

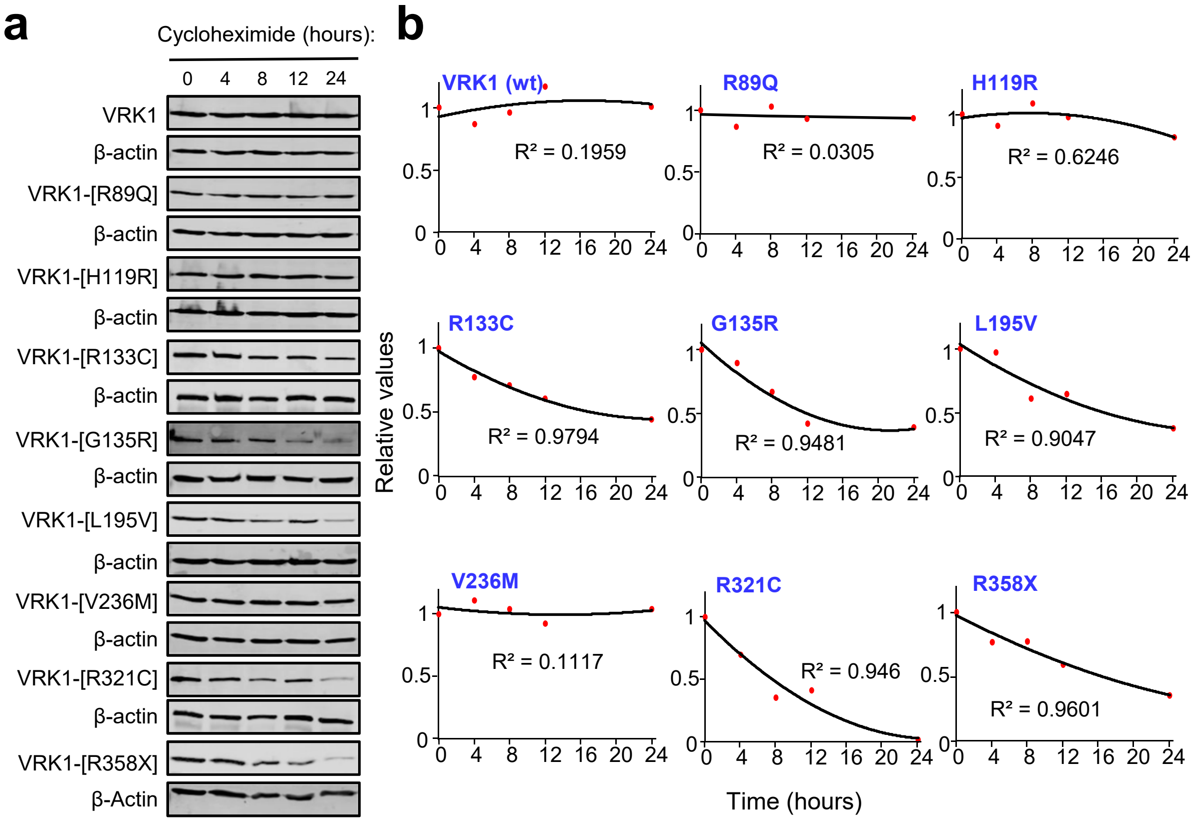

Supplementary Figure S7. **Stability of VRK1 pathogenic variants**. **a.** Western blots showing each individual VRK1 pathogenic variant at different time points, up to twenty-four hours, after cycloheximide addition. **b.** Polynomic regression analysis of each individual VRK1 pathogenic variant. Values are relative to the starting point.

a

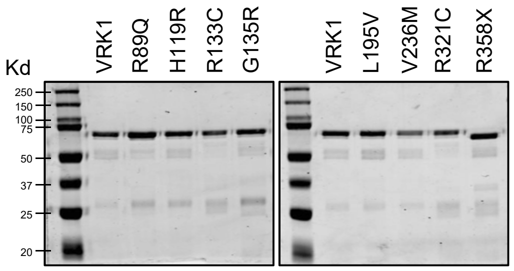

b

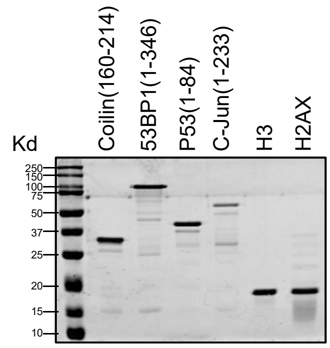

Supplementary Figure S8. **Proteins used in the kinase and stability assays. a**. VRK1 and its different pathogenic variants. The proteins were detected with Coomassie blue staining. **b.** Phosphorylation substrates used in kinase assays either radiactive or for detection with antibodies. The proteins were detected with Coomassie blue staining.

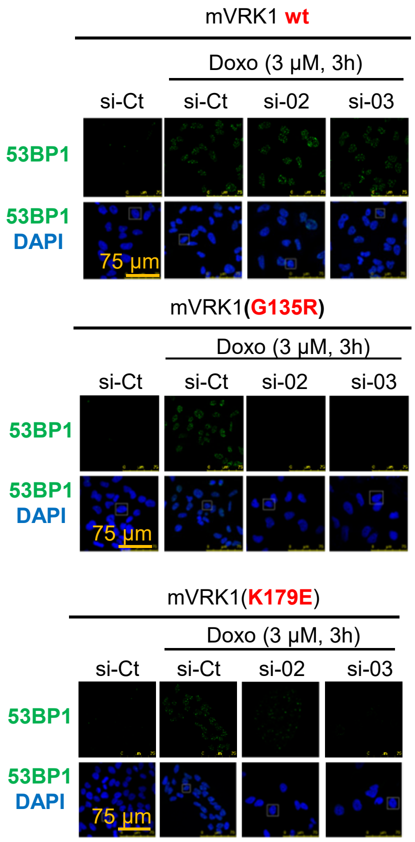

Supplementary Figure S9. **Effect of the mVRK1(G135R) variant on 53BP1 foci induced by DNA damage**. The mVRK1 wild type and the kinase-dead mVRK1(K179E) were used as positive and negative controls. The selected cell shown in Figure S8 is indicated by a square box. Hela cells expressing the murine VRK1 (mVRK1) wt (top), G135R (center) and kinase-dead (K179E) (bottom) were depleted of endogenous human VRK1 with two different siRNA followed by treatment with doxorubicin to induce 53BP1 foci. The quantification of the effect is shown in the graph to the right. * $p < 0.05$, ** $p < 0.005$, *** $p < 0.0005$. The expression of the proteins detected in western blot is shown in Figure 7.
